# Childhood maltreatment, adulthood obesity and incident type 2 diabetes: a retrospective cohort study using UK Biobank

**DOI:** 10.1038/s41366-024-01652-x

**Published:** 2024-10-15

**Authors:** Tamta Nadaraia, Ed Whittaker, Indira Kenyon, Jirapitcha Boonpor, Ziyi Zhou, Shinya Nakada, Ike Dhiah Rochmawati, Carlos Celis-Morales, Joey Ward, Naja Hulvej Rod, Jill P. Pell, Helen Minnis, Thomas Hehlmann, Frederick K. Ho, Daniel Mackay

**Affiliations:** 1 School of Health and Wellbeing, University of Glasgow, Glasgow, UK; 2 Department of Human and Health Sciences, University of Bremen, Bremen, Germany; 3 NHS Greater Glasgow and Clyde, Glasgow, UK; 4 School of Cardiovascular and Metabolic Health, University of Glasgow, Glasgow, UK; 5 Faculty of Public Health, Kasetsart University, Sakon Nakhon, Thailand; 6 Faculty of Pharmacy, University of Surabaya, Surabaya, Indonesia; 7 Human Performance Lab, Education, Physical Activity and Health Research Unit, University Católica del Maule, Talca, Chile; 8 Centro de Investigación en Medicina de Altura (CEIMA), Universidad Arturo Prat, Iquique, Chile; 9 Copenhagen Health Complexity Center, University of Copenhagen, Copenhagen, Denmark

**Keywords:** Type 2 diabetes, Epidemiology, Obesity

## Abstract

**Background:**

This study aims to explore the association of childhood maltreatment with obesity and type 2 diabetes (T2D) in adulthood, and whether obesity is a mediator of the latter.

**Methods:**

In a retrospective cohort study using UK Biobank data, participants recalled childhood maltreatment. Linear regression, logistic regression, and Cox proportional hazard models were used to investigate the associations with body mass index (BMI), obesity, and T2D, adjusted for sociodemographic factors. Decomposition analysis was used to examine the extent to which T2D excess risk was attributed to BMI.

**Results:**

Of the 153,601 participants who completed the childhood maltreatment questions, one-third reported some form of maltreatment. Prevalence of adult obesity and incidence of T2D were higher with the number of reported childhood maltreatment types. People who reported ≥3 types of childhood maltreatment were at higher risk of obesity (OR 1.55, 95% CI 1.47–1.63) and incident T2D (HR 1.65, 95% CI 1.52–1.80). Excess T2D risk among those reporting maltreatment could be reduced by 39% if their BMI was comparable to participants who had not been maltreated, assuming causality.

**Conclusions:**

People who recalled maltreatment in childhood are at higher risk of T2D in adulthood, partly due to obesity.

## Introduction

Obesity has emerged as a global epidemic, contributing significantly to the disease burden in both developed and developing nations [1]. Over the past two decades, the prevalence of obesity has risen dramatically, doubling in adults and children, and tripling in adolescents [2]. Excessive body fat accumulation poses immediate health risks and can lead to severe long-term consequences. These include the development of type 2 diabetes (T2D), coronary heart disease, osteoarthritis, and certain cancers [3].

In the United Kingdom, obesity contributes to approximately 8.7% of all deaths, resulting in an estimated 9000 premature fatalities annually [4]. Due to its increasing prevalence and substantial economic and health-related costs, obesity is now recognised as a critical public health issue [5]. Obesity significantly impacts physical health, mental well-being, and health-related quality of life. It imposes considerable direct and indirect costs on both individuals and healthcare systems [6]. Notably, T2D stands out as one of the most financially burdensome public health consequences of obesity, especially in industrialised nations, as well as having a significantly negative impact on individuals health [7].

The complex determinants of obesity include individual factors, including genetics [8], and multifaceted interactions with our environment. These environmental factors encompass aspects like food supply, dietary behaviours, family-work dynamics, socioeconomic status, urban design, and public policies [6]. Importantly, a growing body of evidence [9] suggests a link between adverse childhood experiences and an increased risk of obesity and T2D in adulthood [10, 11]. Among these adverse experiences, childhood maltreatment, which includes abuse and neglect, is particularly severe. Each year 4–16% of children are physically abused and 10% are neglected or psychologically abused in high-income countries [12].

A recently proposed model for obesity development places focus on the impact of social adversity within the family, including abuse and maltreatment [9]. However, the associations between childhood maltreatment and T2D outcomes remain underexplored, with only limited research investigating this connection [13]. Methods differ for measuring childhood maltreatment, some focusing on the impact of specific maltreatment subtypes, rather than the potential cumulative impact. In addition, T2D is often a self-reported outcome in these studies [14, 15] increasing risk of information bias. Potential mediators between childhood maltreatment and T2D already explored in the literature, include mental ill-health such as depression [16], and altered neuroendocrine stress responsivity, caused by chronic trauma and stress [17, 18].

This study aims to investigate the associations of childhood maltreatment (which includes sexual abuse, physical, verbal, and emotional abuse, as well as emotional neglect) with obesity prevalence and risk of T2D in adulthood within the UK population. Crucially, this study will also examine whether obesity lies on the causal pathway between childhood maltreatment and T2D.

## Methods

### Study design and participants

This retrospective cohort study utilised data from UK Biobank (baseline assessment in 2007–2010), with participants recalling child maltreatment in an online mental health questionnaire in 2017. Follow-up for type 2 diabetes incidence started from the completion of mental health questionnaire in 2017 till the end of 2022.

The UK Biobank is a large biomedical database established in 2007, with various follow-up (including mental health questions in 2017), and linkage to clinical data (e.g. hospital admission and death). The recruitment phase and baseline assessments spanned from 2007 to 2010 and enroled 502,506 participants aged 37–73 years from the general population who attended assessment centres across England, Scotland, and Wales. At the baseline assessment, participants engaged in a structured assessment process, which included the completion of a self-administered touch-screen questionnaire, face-to-face interviews, and physical measurements such as height, weight, and blood pressure, administered by trained personnel. Self-reported information included ethnicity, educational attainment, sleep duration, television viewing time, smoking status, and alcohol consumption. Height and weight assessments were conducted by trained staff, with height measured to the nearest centimetre using a Seca 202 stadiometer and weight recorded to the nearest 0.1 kg using a Tanita BC-418 body composition analyzer. Physical activity levels were self-reported using the validated International Physical Activity Questionnaire [19].

All participants provided written informed consent before enrolment in the UK Biobank, which was conducted in accordance with the Declaration of Helsinki. The UK Biobank study, and the sharing of anonymized data with the research community, was approved by the North West Multi-centre Research Ethics Committee (REC reference: 12/NW/03820). All methods were performed in accordance with the relevant guidelines and regulations.

### Childhood maltreatment

The assessment of childhood maltreatment was carried out through a web-based questionnaire in August 2017 [20]. The participants aged 45–80 (median 64) years when they completed the questionnaire. A total of 339,229 participants who had provided an email address were invited to participate, with approximately half of them (*n* = 157,348) completing the online questionnaire. The questionnaire covered various aspects of their thoughts and emotions, encompassing experiences from their formative years, such as instances of childhood sexual abuse, physical abuse by a family member, feelings of love during childhood, perceptions of being disliked by a family member during childhood, and whether they had someone available to accompany them to medical appointments when necessary.

Respondents who completed the questionnaire generally were younger, more likely to be female and of white ethnicity, and exhibited healthier lifestyles when compared to non-respondents [21]. The web-based questionnaire featured the Childhood Trauma Screener (CTS) [22]. This screener was derived from a condensed version of the Childhood Trauma Questionnaire (CTQ) [23]. The questionnaire did not specify the age at which maltreatment occurred but used general description (‘when I was growing up’) to ascertain childhood maltreatment. It consists of a 5-point Likert scale for each of five types of child maltreatment, physical abuse, physical neglect, emotional abuse, emotional neglect, and sexual abuse. The CTQ is a widely used instrument for measuring reported child maltreatment and has been validated against actual records of abuse and neglect [24]. For this study we used validated cut-off values to identify participants to have specific types of maltreatment [22]. Participants were then categorised according to the number of types of child maltreatment experienced (0, 1, 2 or ≥3) as the primary exposure. This approach allowed us to analyse the impact of the multiplicity of childhood maltreatment that individuals encountered. In addition, childhood maltreatment was also characterised as binary (0 vs. ≥1) and ordinal (exact number of types) variables in analyses.

### Outcome ascertainment

Body mass index (BMI) and obesity were both computed from participants’ height and weight recorded at baseline assessment (2007–2010). For BMI, weight was divided by height squared. The study’s main outcome – obesity - was defined using the WHO BMI category ( ≥ 30.0 kg/m²).

T2D was measured over follow up by individually linking UK Biobank assessment data with death, hospital, and primary care records which included ICD-10 codes (E11) or READ codes. Details are published elsewhere [25]. The start of follow-up was the date of the first assessment visit. Hospital admission data and mortality data were available until October, August, and May 2022 for England, Scotland, and Wales respectively. There were 6289 participants who developed T2D prior to the start of follow-up and were excluded in the T2D analysis.

### Statistical analyses

Descriptive characteristics for the cohort, by number of child maltreatment types, were firstly summarised. For continuous variables the mean and standard deviation (SD) was presented. Linear and logistic regression analyses were then used to examine the associations between child maltreatment and BMI and obesity respectively. For the outcome of BMI, the results were expressed as unstandardised coefficients (Beta) which represented the difference in BMI associated with each additional type of childhood maltreatment. We employed a logistic regression model to investigate the binary outcome of obesity. The odds ratios (OR) were calculated to determine the odds of having obesity for individuals who experienced specific types of childhood maltreatment compared to those who experienced none. To explore the relationship between childhood maltreatment and incident T2D, we used Cox proportional hazard models which consider both the event status and time-to-event. Hazard ratios (HRs) and their 95% confidence intervals (CIs) were reported. Our analyses adjusted for potential confounding variables, including age at baseline assessment, sex, ethnicity, deprivation index, and education level. We also additionally adjusted for the potential mediators, depression, and Post Traumatic Stress Disorder (PTSD), in a sensitivity analysis to determine if the associations were independent of clinical mental health conditions.

We then investigated the independent associations of different types of childhood maltreatment, including physical abuse, emotional abuse, sexual abuse, physical neglect, and emotional neglect, with the outcomes. These childhood maltreatment variables were mutually adjusted in the models.

Lastly, we conducted a four-way decomposition analysis to examine the causal pathway from maltreatment, via BMI, to T2D. The formal definitions and formulas could be found in previous literature [26]. The analysis decomposes the excess risk of T2D in people who reported maltreatment into four components: pure mediation via BMI, interacted mediation via BMI, pure interaction with BMI, and unrelated to BMI. The primary metrics of interest are the proportion attributed to interaction and mediation (including interacted mediation and pure mediation). These two combined indicates the excess risk due to maltreatment that could be hypothetically eliminated if BMI could be equivalised. In addition to the sociodemographic covariates, we also conducted a sensitivity analysis to adjust for lifestyle factors (smoking status, alcohol consumption, physical activity, sedentary behaviour, and sleep duration) as exposure induced confounders (i.e. confounding between mediator and outcome originated from the exposure). The causal diagram for this is shown in Supplementary Fig. 1.

All analyses were conducted using R Statistical Software (version 4.3.1) with packages *survival* and *CMAverse*.

## Results

This study included a cohort of 153,601 UK Biobank participants, with a mean (SD) age of 55.34 (7.74) years, and comprising 59.75% women at baseline. Among these individuals, one-third (*n* = 51,181) reported experiencing at least one form of child maltreatment, with 12.95% reporting multiple types. People who reported higher numbers of maltreatment types were younger, more likely to be female, less likely to be of white ethnicity, reported less physical activity, consumed more alcohol, spent more time watching television, and were less likely to be never smokers (Table 1). Of all types of maltreatments, emotional neglect was most prevalent (22.1%; Supplementary Table 1), followed by emotional abuse (9.3%), and sexual abuse (8.8%). Among people who reported multiple maltreatments, emotional neglect (88.0%) was also the most prevalent, followed by emotional abuse (58.8%) and physical abuse (47.1%).

**Table 1:** Participant characteristics by number of childhood maltreatment types.

Characteristic	Number of child maltreatment types			
	None	One	Two	Three or more
Total *n* (%)	102,420 (66.68%)	31,279 (20.36%)	11,759 (7.66%)	8143 (5.30%)
Mean (SD) age, years	56.16 (7.71)	55.80 (7.74)	55.23 (7.80)	54.18 (7.71)
**Sex**				
Female	56,453 (55%)	17,320 (55%)	7159 (61%)	5564 (68%)
Male	45,963 (45%)	13,958 (45%)	4600 (39%)	2578 (32%)
**Ethnicity**				
White	100,084 (98%)	30,068 (96%)	11,145 (95%)	7,478 (92%)
South Asian	660(0.6%)	328 (1.0%)	146 (1.2%)	127 (1.6%)
Black	455 (0.4%)	238 (0.8%)	155 (1.3%)	220 (2.7%)
Chinese	144 (0.1%)	108 (0.3%)	54 (0.5%)	43 (0.5%)
Mixed	362 (0.4%)	200 (0.6%)	103 (0.9%)	127 (1.6%)
Any other	715 (0.7%)	337 (1.1%)	156 (1.3%)	148 (1.8%)
**Mean (SD) deprivation index**	−1.89 (2.72)	−1.55 (2.88)	−1.26 (3.03)	−0.78 (3.25)
**Education level**				
College or University degree	47,643 (47%)	13,897 (45%)	4961 (42%)	3232 (40%)
A levels/AS levels or equivalent	13,928 (14%)	4143 (13%)	1534 (13%)	1036 (13%)
O levels/GCSEs or equivalent	20,107 (20%)	6137 (20%)	2365 (20%)	1650 (20%)
NVQ or HND or HNC or equivalent	4863 (4.8%)	1678 (5.4%)	653 (5.6%)	521 (6.4%)
SEs or equivalent	3457 (3.4%)	1225 (3.9%)	537 (4.6%)	418 (5.2%)
Other professional qualifications eg: nursing, teaching	5058 (5.0%)	1609 (5.2%)	603 (5.2%)	462 (5.7%)
None of the above	6454 (6.3%)	2279 (7.3%)	970 (8.3%)	742 (9.2%)
Prefer not to answer	278 (0.3%)	103 (0.3%)	58 (0.5%)	35 (0.4%)
**Sleep duration**				
Less than 6 h	3113 (3.0%)	1319 (4.2%)	658 (5.6%)	656 (8.1%)
Between 6–9 h	93,359 (91%)	27,919 (89%)	10,276 (87%)	6838 (84%)
More than 9 h	5948 (5.8%)	2041 (6.5%)	825 (7.0%)	649 (8.0%)
**Mean (SD) physical activity MET·min of /week**	2315.96 (2055.72)	2333.09 (2113.53)	2395.20 (2152.74)	2562.44 (2326.98)
**BMI categories**				
Underweight	574 (0.6%)	176 (0.6%)	56 (0.5%)	53 (0.7%)
Normal	39,141 (38%)	11,307 (36%)	4027 (34%)	2555 (31%)
Overweight	43,850 (43%)	13,374 (43%)	4908 (42%)	3328 (41%)
Obesity	18,855 (18%)	6422 (21%)	2768 (24%)	2207 (27%)
**Smoking status**				
Current	6355 (6.2%)	2517 (8.0%)	1119 (9.5%)	1065 (13%)
Never	61,948 (60%)	17,118 (55%)	5928 (50%)	3664 (45%)
Previous	34,117 (33%)	11,644 (37%)	4712 (40%)	3414 (42%)
**Alcohol drinking** > **14 units/week**	40,717 (40%)	12,351 (39%)	4300 (37%)	2648 (33%)
**Mean (SD) hours of TV viewing/day**	2.45 (1.38)	2.48 (1.44)	2.53 (1.50)	2.59 (1.58)
**T2D**	4979 (4.9%)	1768 (5.7%)	783 (6.7%)	624 (7.7%)

A higher number of maltreatments were associated with a higher odds of obesity (Table 2). Participants who reported more than three types of childhood maltreatment demonstrated the highest odds of obesity in adulthood (OR 1.55 [95% CI 1.47–1.63], *P* < 0.001), followed by those participants who reported two experiences (OR 1.31 [95% CI 1.25–1.37], *P* < 0.001) and one type of maltreatment (OR 1.12 [95% CI 1.08–1.16], *P* < 0.001) compared to those who did not report maltreatment. The risk of developing T2D in adulthood was also associated significantly with the number of types of childhood maltreatment ( ≥ 3 types: HR 1.65 [95% CI 1.52–1.80], *P* < 0.001; 2 types: HR 1.37 [95% CI 1.27–1.47], *P* < 0.001) (Table 2). These associations remained when the model was adjusted for depression and PTSD ( ≥ 3 types and obesity: OR 1.27 [95% CI 1.20–1.34], *P* < 0.001; ≥3 types and T2D: HR 1.28 [95% CI 1.16–1.48], *P* < 0.001) (Supplementary Table 2).

**Table 2:** Association of the number of types of childhood maltreatment with BMI, obesity, and incident T2D.

	BMI			Obesity			Incident T2D		
	Beta	95% CI	p-value	OR	95% CI	*p*-value	HR	95% CI	*p*-value
Childhood maltreatment (Yes vs. no)	0.44	0.40, 0.50	<0.001	1.23	1.19, 1.26	<0.001	1.23	1.18, 1.29	<0.001
Per number of maltreatment types	0.30	0.28, 0.33	<0.001	1.14	1.12, 1.15	<0.001	1.15	1.13, 1.17	<0.001
Number of maltreatment types									
0	0	Reference		1	Reference		1	Reference	
1	0.24	0.18, 0.30	<0.001	1.12	1.08, 1.16	<0.001	1.14	1.08, 1.20	<0.001
2	0.59	0.50, 0.67	<0.001	1.31	1.25, 1.37	<0.001	1.37	1.27, 1.47	<0.001
≥3	1.10	0.98, 1.20	<0.001	1.55	1.47, 1.63	<0.001	1.65	1.52, 1.80	<0.001

CI Confidence Interval, OR Odds Ratio, HR Hazard Ratio; Adjusted for age, sex, ethnicity, deprivation, education.

Examining specific maltreatment types, participants who reported physical abuse (beta 0.94 [95% CI 0.84–1.0], *P* < 0.001) and physical neglect (beta 0.55 [95% CI 0.45–0.65], *P* < 0.001) during childhood exhibited a higher BMI (Table 3). The associations with obesity were larger for physical abuse (OR 1.42 [95% CI 1.36–1.49], *P* < 0.001) and neglect (OR 1.23 [95% CI 1.16–1.29], *P* < 0.001) than for sexual abuse, emotional abuse, and neglect. Regarding T2D, participants who reported physical neglect (HR 1.45 [95% CI 1.33–1.57], *P* < 0.001), physical abuse (HR 1.36 [95% CI 1.26–1.47], *P* < 0.001), or emotional neglect (HR 1.10 [95% CI 1.04–1.17], *P* < 0.001) in childhood were at higher risk of T2D in adulthood.

**Table 3:** Association between the types of childhood maltreatment and metabolic outcomes.

	BMI			Obesity			T2D		
	Beta	95% CI	*p*-value	OR	95% CI	*p*-value	HR	95% CI	*p*-value
Physical Abuse	0.94	0.84, 1.00	<0.001	1.42	1.36, 1.49	<0.001	1.36	1.26, 1.47	<0.001
Emotional Abuse	0.09	0.00, 0.17	0.06	1.10	1.05, 1.16	<0.001	0.99	0.92, 1.08	0.90
Sexual Abuse	0.31	0.23, 0.39	<0.001	1.17	1.12, 1.22	<0.001	1.03	0.96, 1.11	0.40
Physical Neglect	0.55	0.45, 0.65	<0.001	1.23	1.16, 1.29	<0.001	1.45	1.33, 1.57	<0.001
Emotional Neglect	0.06	0.00, 0.12	0.06	1.03	0.99, 1.06	0.11	1.10	1.04, 1.17	<0.001

CI Confidence Interval, OR Odds Ratio, HR Hazard Ratio, Adjusted for other childhood maltreatment types, age, sex, ethnicity, deprivation, education.

Results of the four-way decomposition analysis is shown in Table 4. Consistent with the regression analyses, each additional type of childhood maltreatment was associated with 17% higher T2D risk (HR = 1.17) even after adjusted for exposure-induced confounders in Model 2. Of the excess risk, 8.4% was attributed to the interaction with BMI and 31.1% was attributed to mediation via BMI. Totally, 39.4% of the excess risk due to childhood maltreatment could be attributed to higher BMI.

**Table 4:** Mediation analysis on the association between childhood maltreatment and T2D via BMI.

	Model 1				Model 2			
	Estimate	LCI^a^	UCI^b^	*p*-value	Estimate	LCI^a^	UCI^b^	*p*-value
Total effect, HR	1.16	1.14	1.18	<0.001	1.17	1.14	1.20	<0.001
Total natural direct effect, HR	1.11	1.09	1.13	<0.001	1.12	1.09	1.14	<0.001
Pure natural indirect effect, HR	1.05	1.04	1.05	<0.001	1.05	1.04	1.05	<0.001
% of excess risk attributed to:								
Interaction with BMI	9.7	1.1	19.0	0.02	8.4	0.8	17.0	0.03
Mediation via BMI	32.4	27.6	39.0	<0.001	31.1	26.0	36.1	<0.001
Total via BMI	42.2	32.8	52.1	<0.001	39.4	32.9	49.2	<0.001

Model 1 adjusted for age, sex, ethnicity, deprivation, education; Model 2 additionally adjusted for physical activity, TV viewing, sleep duration, alcohol consumption and smoking status as exposure-induced mediator-outcome confounders.

^a^LCI - Lower 95% confidence interval.

^b^UCI - Upper 95% confidence interval.

## Discussion

The findings presented in this study highlight people who have reported childhood maltreatment were more likely to have obesity and develop T2D in later adulthood. The analysis also shows that a considerable portion, almost 40%, of the association between childhood maltreatment and adult T2D, is mediated through obesity.

### Comparison with existing literature

The findings of this study largely corroborated with existing literature [27] but also meaningfully extend our understanding on the relationship. A prospective longitudinal study investigating the influence of various childhood adversities on the development of obesity and T2D in adulthood showed that stressful emotional experiences during childhood were associated with an elevated risk of obesity and, consequently, increased susceptibility to T2D [28]. A UK-based cross-sectional study investigated the associations between the number of ACEs and self-reported T2D, showing a steep gradient in risk of T2D with 4 or more ACEs, as well as similar results with related conditions including cardiovascular disease and stroke [29].

There have been more studies on childhood maltreatment and obesity in childhood or adolescence, which showed complex differential associations by sex [30] and by types of maltreatment [31]. The literature on adulthood obesity is also heterogeneous. A retrospective cohort study explored reports of different forms of abuse, encompassing sexual, verbal, physical abuse, as well as the fear of physical abuse, and their associations with adult obesity. Associations were reported between all forms of abuse and higher body weight in adulthood. Consistent with our findings, physical abuse emerged as the most important contributor to a higher body weight and adult obesity [32]. Interestingly, the US CARDIA study has found no association between exposure to abuse in childhood and obesity [33]. The inconsistency could be, in part, be attributed to the measurements. The abuse questions of the CARDIA study focused on only physical and psychological abuse, while this study included also neglect and sexual abuse. That study also only focused on incidence of obesity between 18–30 years and 48–60 years. while this study examined prevalence at 37–70 years. There is, however, a small longitudinal study that also showed association between early life trauma and development of adult obesity [34]. Importantly, none of these studies explicitly modelled the mediation pathways via BMI, which provides useful insights in developing relevant leverage points for intervention.

### Causal pathways

In this study we demonstrated a considerable mediating role of BMI between childhood maltreatment and T2D. Several studies have explored the mechanism linking childhood maltreatment and obesity. A sequence of models that tested mediation effects indicated that stress-induced overeating among respondents with problematic histories of violence explained, in part, their higher risk of adult obesity [35]. Other studies [17] suggest depression as a potential mediator, after demonstrating that the association between childhood maltreatment and T2D was stronger among those with depression [16]. Interestingly, our study has shown that even when clinical depression was adjusted, the association between maltreatment and T2D remained.

### Limitations

The study had some limitations. The study relied on self-report of childhood maltreatment. This raises the possibility of recall bias, and underreporting given the sensitivity of the topic. However, abuse is often also underestimated by objective sources due to underreporting. Participants who had already developed T2D prior to the start of the study, were excluded. A recent study has shown maltreatment to affect T2D earlier in life, with participants aged 16 years at baseline [17]. Owing to the timing of the obesity measurement, it was necessary to exclude those participants. It should be noted that our studies finding could therefore be subject to truncation bias, leading to more conservative effect sizes. The study sample was obtained from UK Biobank, which is not representative of the general population, including a relatively healthy and more socioeconomically advantaged group. Nonetheless previous studies have shown that findings from UK Biobank were comparable to population representative studies [36]. Although the study takes into account several potential confounding variables, it may not consider all relevant factors that could influence the association between childhood maltreatment and obesity and T2DM, such as attention deficit hyperactivity disorder (ADHD) [37]. Therefore, as with all observational studies, causality cannot be inferred even though it was assumed in the mediation analysis. Importantly, there was no information on the source of abuse, intensity, duration, and timing, as well as the onset of obesity, all of which could help further delineate the associations of childhood maltreatment with obesity and T2D better. Future studies should investigate these.

### Implications

This study contributes to the increasing evidence linking childhood maltreatment to adverse metabolic health outcomes in adulthood. It highlights the need for a life-course approach to public health that considers early childhood experiences and their potential impact on health in adulthood [9]. Current literature on interventions to improve health outcomes is very limited with respect to neurobiological/physical outcomes, with most focus being on mitigating psychological harms [38]. Future research should cover a wide range of dimensions, from understanding the underlying mechanisms that include both environmental exposures and individual factors, to developing effective prevention and intervention strategies [39], including addressing neurobiological/physical health outcomes. Research should also ensure to include both environmental exposures and individual factors contributing to obesity, including neurodevelopment and appetite. In addition, research should be integrated into the development of health policies and strategies aimed at preventing childhood maltreatment and its long-term consequences. This may include addressing social determinants, strengthening access to mental health and domestic abuse services, and promoting safe, stable, and supportive environments and relationships for children and families.

## Conclusions

Childhood maltreatment recall, especially physical abuse and neglect, was associated with obesity and T2D in adulthood. Prevention of childhood maltreatment could be an important element in managing obesity and T2D. Among people who were maltreated, obesity is a modifiable mediator of the association with T2D and therefore a potential intervention target.

## Summary table

### What is already known about this subject?

- Obesity is known to be a multifactorial condition, where risk is not fully explained by established risk factors (e.g. genetics, lifestyle)
- The Developmental Origins of Health and Disease (DOHaD) has postulated early life factors could explain obesity
- Preliminary evidence has shown associations of early life adversity with obesity and type 2 diabetes.

### What are the new findings in your manuscript?

- Prevalence of adult obesity and incidence of T2D were higher in people who reported more childhood maltreatment types.
- People who reported ≥3 types of childhood maltreatment were at higher odds of obesity (OR 1.55, 95% CI 1.47–1.63) and incident T2D (HR 1.65, 95% CI 1.52–1.80).
- Excess T2D risk among those reporting maltreatment could be reduced by 39% if their BMI was comparable to participants who had not been maltreated, assuming causality.

### How might your results change the direction of research or the focus of clinical practice?

- Prevention of childhood maltreatment or intervention of people who had maltreatment could reduce risks of obesity and type 2 diabetes.
- Adulthood obesity could be a consequence of early life adversity that leads to diseases later in life.

## Supplementary information

Supplemental material

## Data Availability

The data supporting the findings of this study were obtained from the UK Biobank, and these data are subject to certain restrictions regarding their availability. They were used under license for the specific purpose of the current study, which means that they are not publicly available for unrestricted use. However, data are available from the authors upon reasonable request and with permission from the UK Biobank.
