# Supplementary material for: Childhood maltreatment, adulthood obesity and incident type 2 diabetes: a retrospective cohort study using UK Biobank
Source: Int J Obes (Lond). 2024 Oct 15;49(1):140–6. doi: 10.1038/s41366-024-01652-x (PMC11683003; doi:10.1038/s41366-024-01652-x)
Supplement: Supplementary file 1 — Supplemental material [file 41366_2024_1652_MOESM1_ESM.docx]

Supplementary Figure 1. Causal diagram from childhood maltreatment to T2D via BMI

Childhood maltreatment

BMI

Incident T2D

Sociodemographic factors

Lifestyle factors

Sociodemographic factors include age, sex, ethnicity, deprivation, education; Lifestyle factors include physical activity, TV viewing, sleep duration, alcohol consumption and smoking status

Supplementary Table 1. Prevalence of types of maltreatments

|  | Overall (n=153,601) | | Among people with ≥2 types of maltreatment (n=19,902) | |
| --- | --- | --- | --- | --- |
|  | N | % | N | % |
| Physical Abuse | 12,325 | 8.0 | 9,379 | 47.1 |
| Emotional Abuse | 14,344 | 9.3 | 11,696 | 58.8 |
| Sexual Abuse | 13,447 | 8.8 | 6,962 | 35.0 |
| Physical Neglect | 8,635 | 5.6 | 5,934 | 29.8 |
| Emotional Neglect | 34,012 | 22.1 | 17,516 | 88.0 |

Supplementary Table 2. Association between the number of types of childhood maltreatment and metabolic outcomes related to depression and PTSD

|  | BMI | | | Obesity | | | Incident T2D | | |
| --- | --- | --- | --- | --- | --- | --- | --- | --- | --- |
| Characteristic | Beta | 95% CI | p-value | OR | 95% CI | p-value | HR | 95% CI | p-value |
| Number of maltreatment types |  |  |  |  |  |  |  |  |  |
| 0 | 0 | Reference |  | 1 | Reference |  | 1 | Reference |  |
| 1 | 0.09 | 0.03, 0.14 | 0.004 | 1.04 | 1.01, 1.08 | 0.009 | 1.04 | 0.98, 1.10 | 0.20 |
| 2 | 0.30 | 0.21, 0.39 | <0.001 | 1.16 | 1.10, 1.22 | <0.001 | 1.17 | 1.08, 1.27 | <0.001 |
| ≥3 | 0.64 | 0.53, 0.74 | <0.001 | 1.27 | 1.20, 1.34 | <0.001 | 1.28 | 1.16, 1.40 | <0.001 |

CI = Confidence Interval, OR = Odds Ratio, HR = Hazard Ratio; Adjusted for age, sex, ethnicity, deprivation, education, depression and PTSD.
